# Improved Glucose and Lipid Metabolism in the Early Life of Female Offspring by Maternal Dietary Genistein Is Associated With Alterations in the Gut Microbiota

**DOI:** 10.3389/fendo.2018.00516

**Published:** 2018-09-04

**Authors:** Liyuan Zhou, Xinhua Xiao, Qian Zhang, Jia Zheng, Ming Li, Miao Yu, Xiaojing Wang, Mingqun Deng, Xiao Zhai, Rongrong Li

**Affiliations:** Key Laboratory of Endocrinology, Department of Endocrinology, Translational Medicine Center, Ministry of Health, Peking Union Medical College Hospital, Peking Union Medical College, Chinese Academy of Medical Sciences, Beijing, China

**Keywords:** dietary genistein, glucose and lipid metabolism, gut microbiota, maternal high-fat diet, female offspring

## Abstract

Maternal over-nutrition can lead to metabolic disorders in offspring, whereas maternal dietary genistein may have beneficial effects on the metabolic health of offspring. Our objective was to determine whether maternal dietary genistein could attenuate the detrimental effects of a maternal high-fat diet on their offspring's metabolism and to explore the role of the gut microbiota on their offspring's glucose and lipid metabolism. C57BL/6 female mice were fed either a high-fat diet without genistein (HF), high-fat diet with low-dose genistein (0.25 g/kg diet) (HF.LG), high-fat diet with high-dose genistein (0.6 g/kg diet) (HF.HG) or normal control diet (Control) for 3 weeks prior to breeding and throughout gestation and lactation. The female offspring in the HF group had lower birth weights and glucose intolerance and higher serum insulin, triacylglycerol (TG) and total cholesterol (TC) levels at weaning compared with the Control group. Offspring from HF.LG dams had increased birth weight, improved glucose tolerance, and decreased fasting insulin, whereas the serum TG and TC levels were decreased in HF.HG offspring in comparison with HF offspring. The significant enrichment of *Bacteroides* and *Akkermansia* in offspring from genistein-fed dams might play vital roles in improving glucose homeostasis and insulin sensitivity, and the significantly increased abundance of *Rikenella* and *Rikenellaceae_RC9_ gut_group* in the HF.HG group may be associated with the decreased serum levels of TG and TC. In conclusion, maternal dietary genistein negates the harmful effects of a maternal high-fat diet on glucose and lipid metabolism in female offspring, in which the altered gut microbiota plays crucial roles. The ability of maternal genistein intake to improve offspring metabolism is important since this intervention could fight the transmission of diabetes to subsequent generations.

## Introduction

Obesity and type 2 diabetes mellitus (T2DM) are highly prevalent and lead to tremendous health and economic burdens. However, the etiology and pathogenesis of obesity and diabetes are still unclear. Since the developmental origins of health and disease (DOHaD) hypothesis was first put forward in the early 1990s (1), a large number of epidemiological investigations (2–4) and animal studies (5, 6) have highlighted the importance of the developmental environment in early life in determining the trajectories of chronic disease in later life, including obesity and T2DM. Numerous recent studies (7, 8) and our previous research (9) have shown that a maternal high-fat diet during pregnancy and lactation can significantly increase the susceptibility of offspring to obesity, glucose intolerance and insulin resistance. Thus, interventions during early life may reset the disease trajectories and prevent the onset and development of diabetes.

Several large epidemiological studies have shown that intake of soy foods and isoflavones are associated with a lower risk of T2DM (10–12). Soy isoflavones have a weak estrogen-like effect, and the main components of soy isoflavones include genistein, diadzein, and glycitein. Genistein has been widely used as a dietary supplement in the United States and has been explored for the potential effects in cognitive function, cancer therapy, and bone and cardiovascular health (13). In recent years, a growing number of studies have shown that genistein improves glucose and lipid metabolism and have demonstrated that genistein intake reduces the levels of blood glucose, triglycerides (TG) and total cholesterol (TC) as well as prevents weight gain, without side adverse effects (14–16). Modulating the hepatic glucose output, enhancing β-cell proliferation, reducing apoptosis, activating the cAMP/PKA signaling pathway and antioxidant effects are all potential mechanisms for the anti-diabetic functions of genistein (13). However, currently, studies exploring the effects of genistein intervention in early life on glucose and lipid metabolism are rare.

During the last few decades, the gut microbiota has become a focus of medical research. Numerous lines of evidence (17–19) have suggested that the gut microbiota plays an important role in glucose and lipid metabolism. Recently, a growing number of human (20) and animal studies (1, 21, 22) have indicated that the gut microbiota is disordered in offspring from obese mothers and high-fat fed dams. Thus, the gut microbiota may play a pivotal role in a poor maternal intrauterine growth environment, programming the offspring to develop metabolic disturbances. Furthermore, an association between the genistein improvement of glucose tolerance and alterations of the gut microbiota has been shown (23). However, investigations into the effects of maternal genistein intervention on the gut microbiota in offspring are limited.

In the current study, we aimed to research the effects of maternal dietary genistein on metabolic health in the early life of female offspring and determine whether maternal genistein intake could reverse the detrimental metabolic effects of a maternal high-fat diet in female offspring. In addition, we explored the role of the gut microbiota on offspring glucose and lipid metabolism.

## Materials and methods

### Animals and study design

Four-week-old C57BL/6 female mice were obtained from the National Institutes for Food and Drug Control (Beijing, China; SCXK-2014-0013). Animals were maintained in controlled animal facilities at a room temperature of 22 ± 2°C with a 12 h light/dark cycle and were fed a normal control diet (AIN-93G diet) with corn oil substituted for soybean oil. After 1 week of environmental acclimatization, dams were randomly divided into four groups and were fed a high-fat diet without genistein (HF, *n* = 6), high-fat diet with low-dose genistein (CAS: 466-72-0, G0272, TCI Development Co., Ltd.) (0.25 g/kg diet) (HF.LG, *n* = 6), high-fat diet with high-dose genistein (0.6 g/kg diet) (HF.HG, *n* = 8) or normal control diet (Control, *n* = 8) for 3 weeks. The soybean oil in the high-fat diet was also substituted by corn oil. The ingredients are shown in Table S1. The high-fat diet included (kcal %): fat, 60%; carbohydrate, 20%; and protein, 20%, with a 5.24 kcal/g energy supply, whereas the control diet contained (kcal %): fat, 15.8%; carbohydrate, 63.9%; and protein, 20%, with a 3.9 kcal/g energy supply.

Female mice were mated to 8-week-old C57BL/6 males and fed a normal diet. The dams were checked for postcopulatory plugs every morning after mating, and the appearance of a plug was recorded as d 0.5 of pregnancy. Females were fed their assigned diet during pregnancy and lactation and had access to food and water *ad libitum*. The litters were all culled to five pups to ensure that there was no nutritional bias between litters. Offspring were weaned at 3 weeks of age. At weaning, all female offspring (*n* = 6–8 per group) were sacrificed. Blood samples were collected from the intraorbital retrobulbar plexus after 10 h of fasting from anesthetized mice, and the uterus and ovaries were removed and weighed; the cecal contents were quickly removed, snap frozen in dry ice, and then stored at −80°C for further analysis. All operations were conducted under chloral hydrate anesthesia, and best efforts were done to minimize suffering. All of the procedures were approved by the animal care and use committee of the Peking Union Medical College Hospital (Beijing, China, SYXC-2014-0029). All of the animal operations were conducted in compliance with the Guide for the Care and Use of Laboratory Animals.

### Measurement of body weight and food intake

The body weights of both the mother and offspring were measured once per week. We measured the 3-day food intake each week by the mother, and their food consumption was estimated by weighing the remaining food.

### Glucose tolerance tests

Oral glucose tolerance tests (OGTT) were performed on both dams and their female offspring at weaning. Mice were fasted for 6 h. Then, a glucose load (2.0 g/kg body weight) was given by gavage. Before (0 min) and at 30, 60, and 120 min after the gavage, the blood glucose (BG) concentration was measured in blood collected from a tail bleed using a Contour TS glucometer (ACCU-CHEK Mobile, Beijing, China). The area under the curve (AUC) of the OGTT was calculated as previously described (9).

### Measurement of serum insulin, triacylglycerol, and total cholesterol levels

The blood samples collected from female offspring at weaning were centrifuged at 3,000 × g for 10 min at 4°C, and the serum was stored in aliquots at −80°C. The serum insulin concentrations were measured using an ELISA kit (80-INSMSU-E01, Salem, NH, USA). Insulin sensitivity was assessed using the homeostasis model assessment of insulin resistance (HOMA-IR). The HOMA-IR was calculated as previously described (9). Serum total cholesterol (TC) (K603-100, kits were from BioVision, Inc., Mountain View, CA, USA) and triacylglycerol (TG) (K622-100, kits were from BioVision Inc., Mountain View, CA, USA) were measured by colorimetric methods.

### Gut microbiota analysis

The gut microbiota was analyzed according to the methods described in our previous publication (24). Microbial DNA was extracted from the cecal content using a QIAamp DNA Stool Mini Kit (Qiagen, Hilden, Germany). The V3-V4 regions of the 16S rRNA gene were amplified using the primers 341F 5′-CCTAYGGGRBGCASCAG-3′ and 806R, 5′-GGACTACNNGG GTATCTAAT-3′. Amplicons were purified using a quick PCR purification kit (Qiagen, Hilden, Germany). Microbial 16S rDNA was sequenced on the Illumina HiSeq 2500 platform (Norcross, GA, USA).

After merging paired-end reads, reads were performed by quality filtering. High quality reads were assigned to operational taxonomic units (OTUs) at the 97% similarity level using UPARSE software (version 7.0.1001) (25), and representative sequences for each OTU were screened using QIIME software (version 1.7.0, Quantitative Insights into Microbial Ecology) (26). Then, the GreenGene Database (27) was used to annotate taxonomic information based on the RDP classifier version 2.2 algorithm (28). The relative abundance of each OTU was analyzed at the phylum, class, order, family, genus and species levels. Alpha and beta diversity were examined using QIIME software (Version 1.7.0) and calculated with R software (Version 2.15.3). For alpha diversity, Chao1, Simpson and the Shannon index were analyzed. For beta diversity, principal component analysis (PCA) plots were performed using both weighted and unweighted UniFrac. In addition, linear discriminant analysis (LDA) of the effect size (LEfSe) and MetaStat were used to determine differences among the groups.

### Statistical analysis

The results are expressed as the mean ± standard error of the mean (S.E.M). The statistics were analyzed by one-way ANOVA and two-way ANOVA, with Tukey and Bonferroni *post-hoc* analyses. Correlations between the relative abundance of bacterial taxa at different taxonomic levels and metabolic parameters were performed by Spearman correlation coefficient test. Correction for correlation analysis by false discovery rate (FDR) with the Benjamini-Hochberg procedure were displayed by R software (Version 2.15.3) with values of < 0.05 considered statistical significance. Analysis of similarities (ANOSIM) was used to test the statistical significance for β diversity. A *p* < 0.05 was considered statistically significant. For the MetaStat analysis, a *q* < 0.05 was considered statistical significance. Prism version 7.0 (GraphPad Software Inc., San Diego, CA, USA) was used for statistical analysis.

## Results

### Characterization of dams

During the 9-week of dietary intervention, the energy intake of dams among the four groups was not significantly different. At weaning, the body weights of high-fat fed dams (HF) were higher than that of dams in the control group (*p* < 0.0001; Table 1). The AUC of the OGTT was significantly larger in dams fed a high-fat diet compared to that of dams in the control group (*p* < 0.05). However, in contrast to the HF group, the body weights and glucose tolerance were not significantly different in dams fed either a high-fat diet with low-dose genistein (HF.LG) or high-fat diet with high-dose genistein (HF.HG). There was no difference among the four groups in regard to litter size, uterus index (weight ratio of uterus to body weight) or ovary index (weight ratio of ovaries to body weight) of dams.

**Table 1:** Dam and liter characteristics.

**Parameters**	**HF**	**HF.LG**	**HF.HG**	**Control**
	(*n* = 6)	(*n* = 6)	(*n* = 8)	(*n* = 8)
Body weight(g)	31.5 ± 0.9	30.7 ± 0.6	30.2 ± 0.6	24.1 ± 0.6^*^
AUC of OGTT(mmol/l∙h)	21.8 ± 1.2	19.7 ± 0.9	21.4 ± 1.2	17.5 ± 0.8^*^
Pups/litter	8.0 ± 0.6	6.8 ± 1.0	7.2 ± 0.5	7.0 ± 0.6
Uterus index (%) to body weight(%)	0.33 ± 0.1	0.42 ± 0.0	0.29 ± 0.1	0.39 ± 0.0
Ovary index (%) to body weight(%)	0.11 ± 0.0	0.13 ± 0.0	0.10 ± 0.0	0.12 ± 0.0

*Data are expressed as means ± S.E.M (n = 6–8/group)*.

* *p < 0.05 vs. HF group. HF, high-fat diet without genistein; HF. LG, high-fat diet with low-dose genistein; HF. HG, high-fat diet with high-dose genistein; Control, normal control diet. Uterus index: weight ratio of uterus to body weight; Ovary index: weight ratio of ovaries to body weight*.

### Birth weight and body weight of offspring

**Figure 1:**
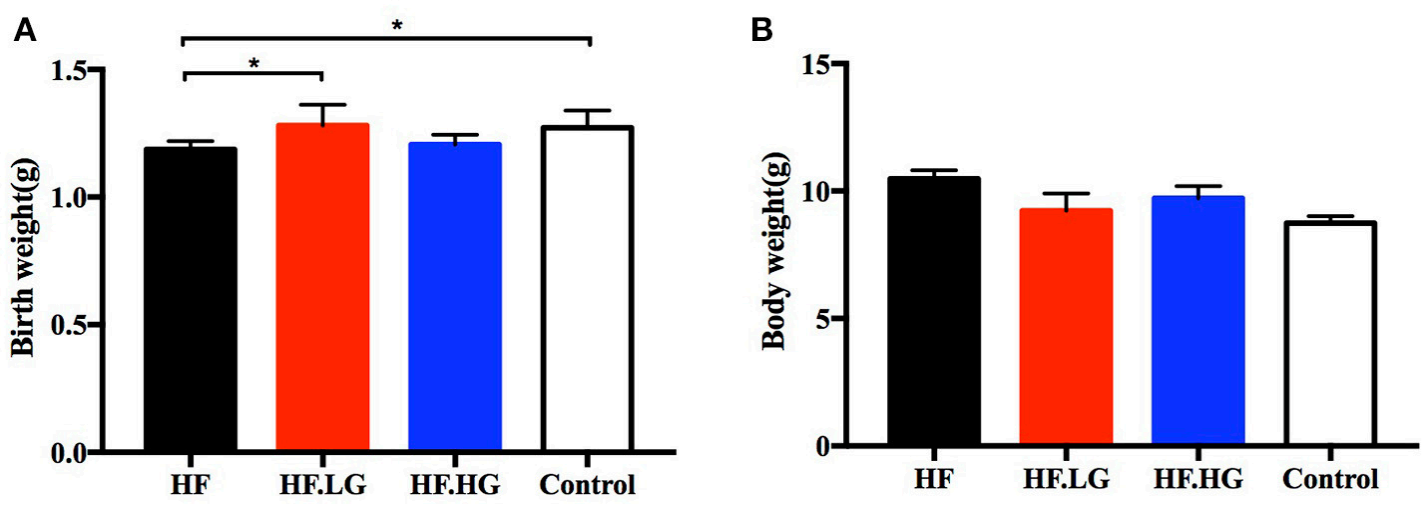
Birth weight and body weight at weaning in offspring. **(A)** Birth weight, and **(B)** Body weight of female offspring at weaning. HF, high-fat diet without genistein; HF. LG, high-fat diet with low-dose genistein; HF. HG, high-fat diet with high-dose genistein; Control, normal control diet. Data are expressed as means ± S.E.M. (*n* = 6–8/group). Mean values were significantly different between other group and the HF group: ^*^p < 0.05.

The birth weights of offspring of high-fat fed dams (HF) were lower than those of offspring of control group dams (*p* < 0.05, Figure 1A). In offspring of high-fat diet with low-dose genistein dams, the birth weights improved and were higher than those of offspring of HF group dams (*p* < 0.05, Figure 1A). However, there was no difference among the four groups in regard to the body weight of female offspring at weaning (Figure 1B).

### Dietary low-dose genistein prevents the deleterious effects of a maternal high-fat diet on glucose metabolism and insulin sensitivity of the offspring

**Figure 2:**
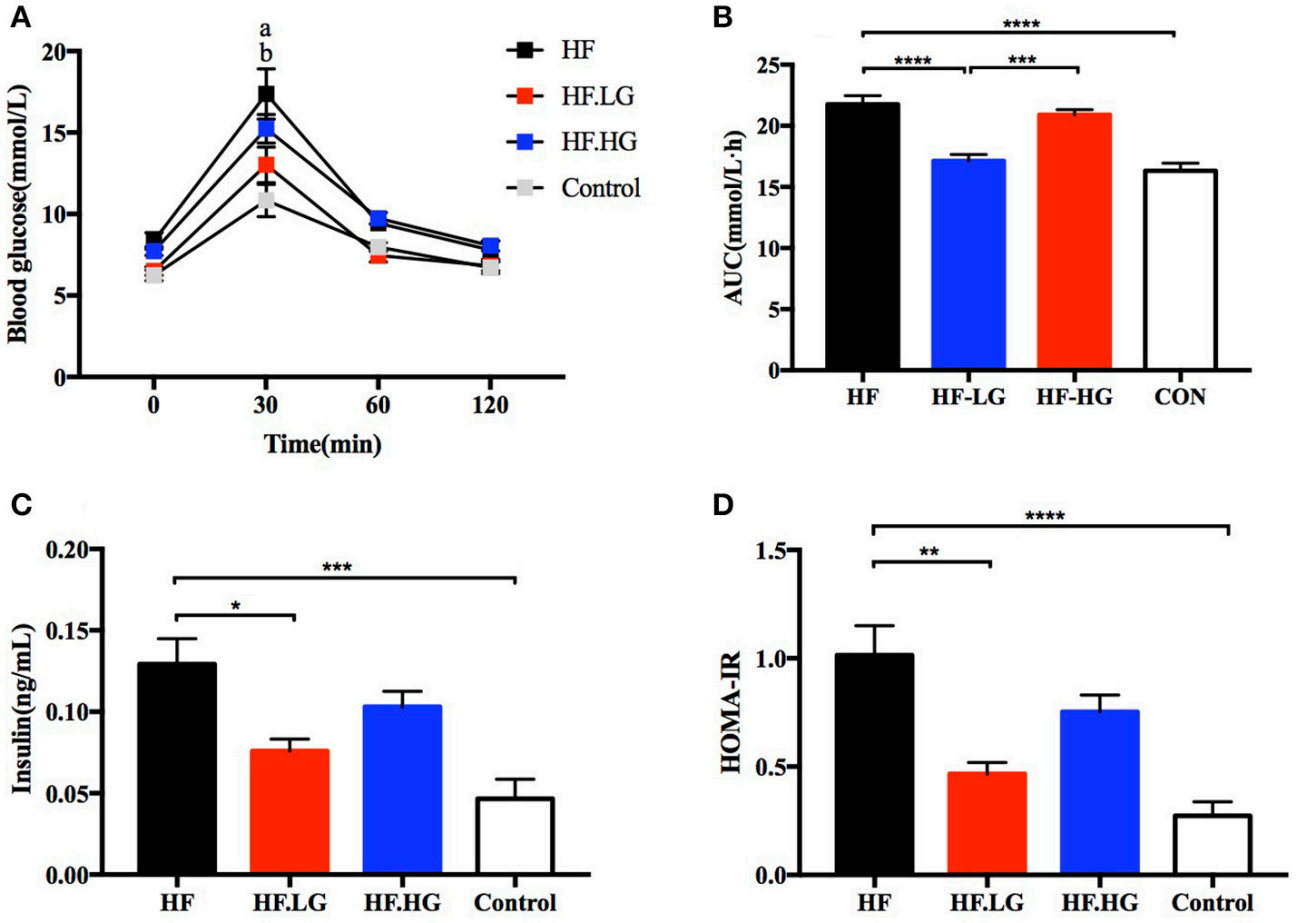
Glucose metabolism of the female offspring at weaning. **(A)** OGTT; **(B)** AUC; **(C)** Serum insulin levels; **(D)** HOMA-IR. HF, high-fat diet without genistein; HF. LG, high-fat diet with low-dose genistein; HF. HG, high-fat diet with high-dose genistein; Control, normal control diet; OGTT, oral glucose tolerance test; AUC, area under the curve; HOMA-IR, the homeostasis model assessment of insulin resistance. Data are expressed as means ± S.E.M. (*n* = 6-8/group). Mean values were significantly different between other group and the HF group: ^*^p < 0.05, ^**^p < 0.01, ^***^p < 0.001, ^****^p < 0.0001; Mean values were significantly different between Control group and the HF group: ‘a' *p* < 0.0001; Mean values were significantly different between HF.LG group and the HF group: ‘b' *p* < 0.001.

At weaning, offspring from high-fat diet fed dams (HF) had impaired glucose tolerance as measured by OGTT compared to offspring from normal control diet fed dams (Control). The blood glucose levels were higher at 30 min (*p* < 0.0001) and the AUC was significantly larger (*p* < 0.0001) for offspring of HF group (Figures 2A,B). To determine whether maternal dietary genistein affected the glucose metabolism of the female offspring, we compared offspring from dams that were fed a high-fat diet with or without genistein. As shown in Figures 2A,B, female offspring of low-dose genistein fed dams (HF.LG) demonstrated a marked improvement in glucose tolerance. The blood glucose levels at 30 min (*p* < 0.0001) and AUC of these female offspring were significantly lower (*p* < 0.0001). Furthermore, to determine whether insulin sensitivity was influenced in female offspring, the level of fasting serum insulin was detected. High-fat fed dams resulted in a significantly higher insulin concentration (*p* < 0.001) and HOMA-IR index (*p* < 0.0001) in offspring at weaning. Low-dose genistein fed dams (HF.LG) led to improved insulin sensitivity in their offspring (Figures 2C,D). However, no significant difference was detected in regard to glucose tolerance and insulin sensitivity between female offspring of HF.HG group and HF group (Figures 2A–D). In addition, no significant differences were identified between offspring of HF.HG and HF.LG group dams in regard to blood glucose levels at different times during the glucose tolerance test, serum insulin levels and HOMA-IR, other than the significantly lower AUC in offspring of HF.LG group (*p* < 0.001).

### Maternal dietary high-dose genistein improves lipid metabolism in the early life of offspring

**Figure 3:**
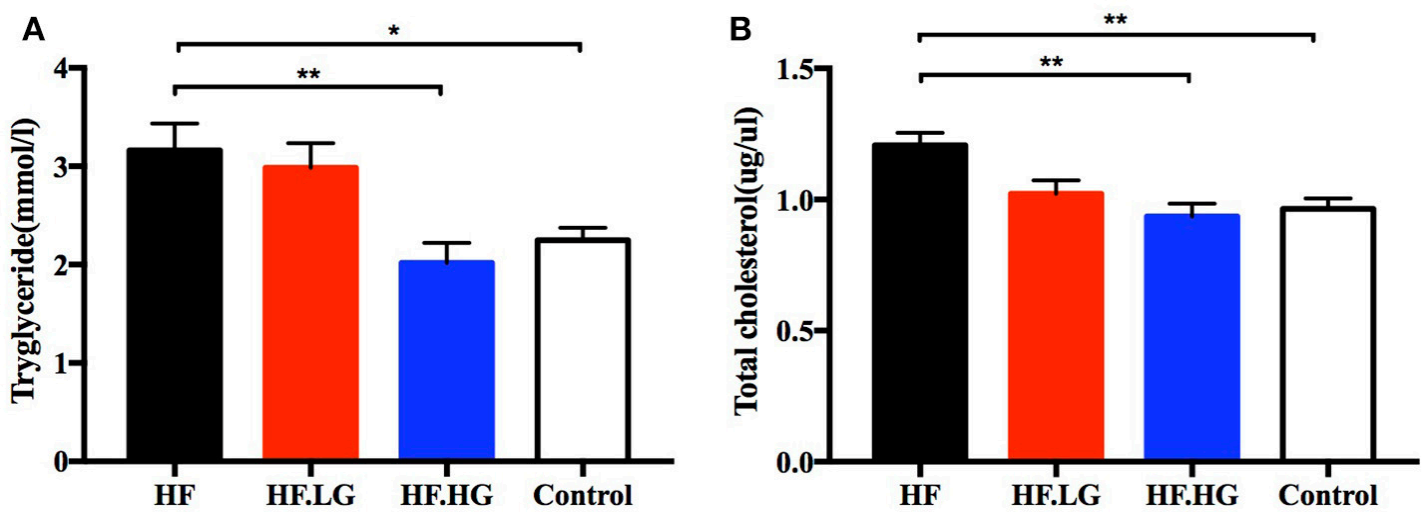
Lipid metabolism of the female offspring at weaning. **(A)** Serum triacyglycerol; and **(B)** serum total cholesterol. HF, high-fat diet without genistein; HF. LG, high-fat diet with low-dose genistein; HF. HG, high-fat diet with high-dose genistein; Control, normal control diet. Data are expressed as means ± S.E.M (*n* = 6-8/group). Mean values were significantly different between other group and the HF group: ^*^p < 0.05, ^**^p < 0.01.

In addition to glucose metabolism, we detected the levels of serum lipids to evaluate the differences between groups regarding lipid metabolism of female offspring at weaning. The levels of serum TG (*p* < 0.05, Figure 3A) and TC (*p* < 0.01, Figure 3B) in the offspring of high-fat fed dams (HF) were higher than those in the offspring of Control group dams. High-dose genistein feeding of dams (HF.HG) resulted in a significant improvement in the serum TG (*p* < 0.01, Figure 3A) and TC levels (*p* < 0.01, Figure 3B) in female offspring. In contrast to the changes in glucose tolerance and insulin sensitivity, there was no significant difference in the serum lipid levels between the offspring of HF.LG group and HF group.

### Effects of maternal dietary genistein on gut microbiota in offspring

**Figure 4:**
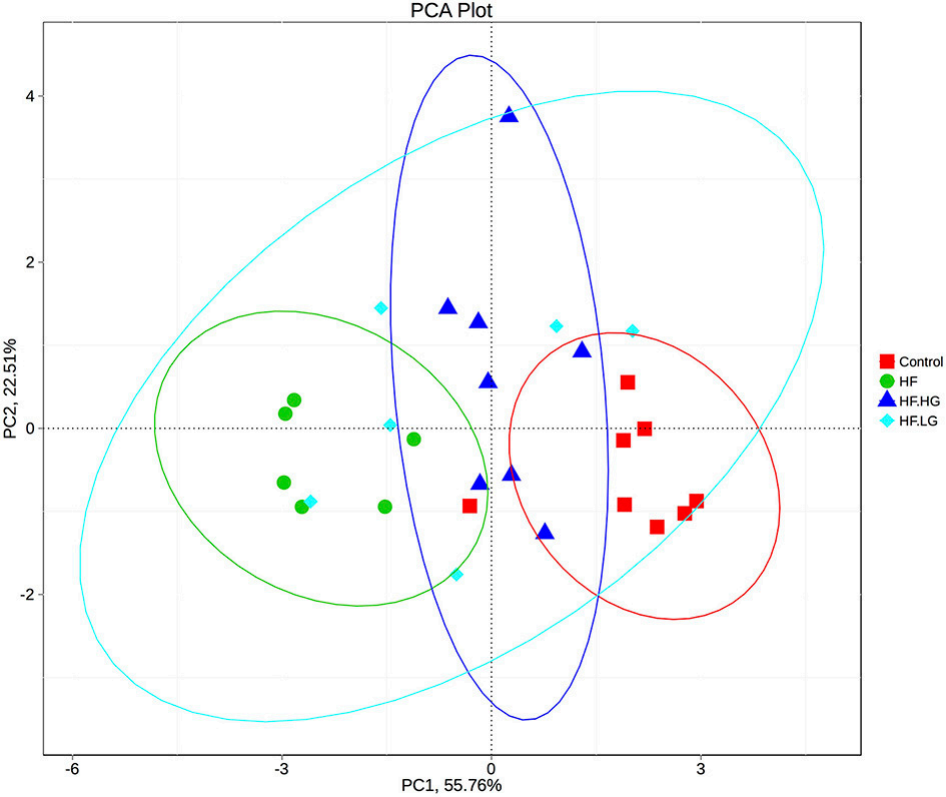
PCA plots of gut communities in the female offspring at weaning (*n* = 6–8/group). HF, high-fat diet without genistein; HF. LG, high-fat diet with low-dose genistein; HF. HG, high-fat diet with high-dose genistein; Control, normal control diet.

**Figure 5:**
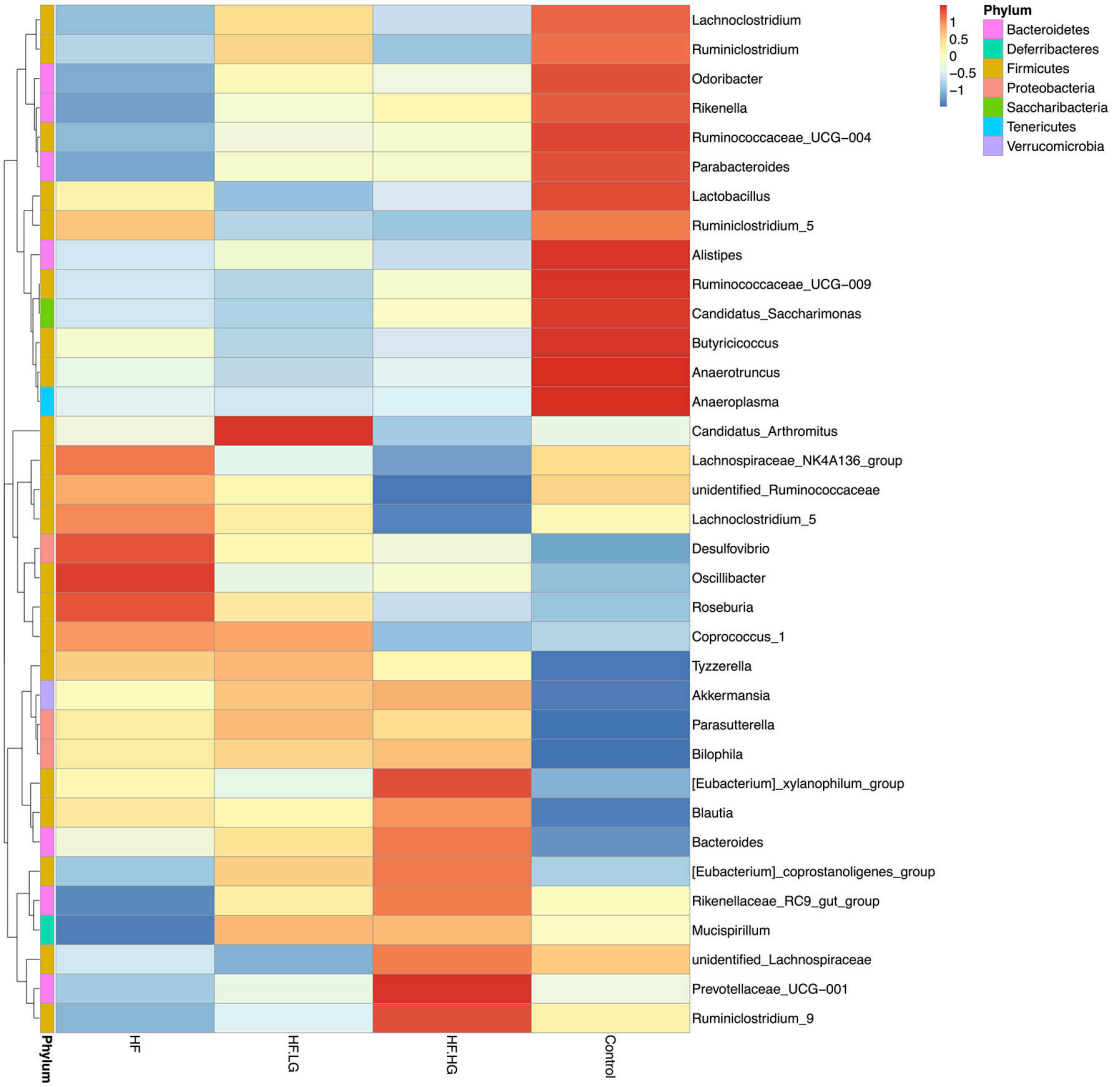
Heat map analyses of abundant genera in each group (*n* = 6–8/group). HF, high-fat diet without genistein; HF. LG, high-fat diet with low-dose genistein; HF. HG, high-fat diet with high-dose genistein; Control, normal control diet.

To explore the mechanisms of maternal dietary genistein improvement of glucose and lipid metabolism in offspring, we analyzed the gut microbiota changes in offspring using 16s rDNA gene sequences. The sequence data in this study has been submitted to the Sequence Read Archive (SRA) database (accession number SRP156380). A total of 1836395 high quality reads were obtained from 28 samples, with an average of 65586 sequences per sample. After clustering at the 97% similarity level, 653 operational taxonomic units (OTU) were identified among the four groups. Alpha diversity analysis showed that there was a parallel community richness (Chao 1) and diversity (Simpson and Shannon index) among groups (Table S2, Figure S1). To compare the overall structure of the gut microbial community, principal component analysis (PCA) was performed to determine differences among groups. As shown in Figure 4, the gut microbial communities were well separated in the HF group compared with both the Control group (*p* < 0.01) and HF.HG group (*p* < 0.05), with 55.76 and 22.51% variations explained by principal component (PC) 1 and PC2, respectively. The results were supported by ANOSIM and showed that there significant differences in the microbial structure were caused by the maternal diets during pre-pregnancy, pregnancy and lactation. This result was also verified by a heatmap according to the bacterial genus level among the four groups (Figure 5).

**Figure 6:**
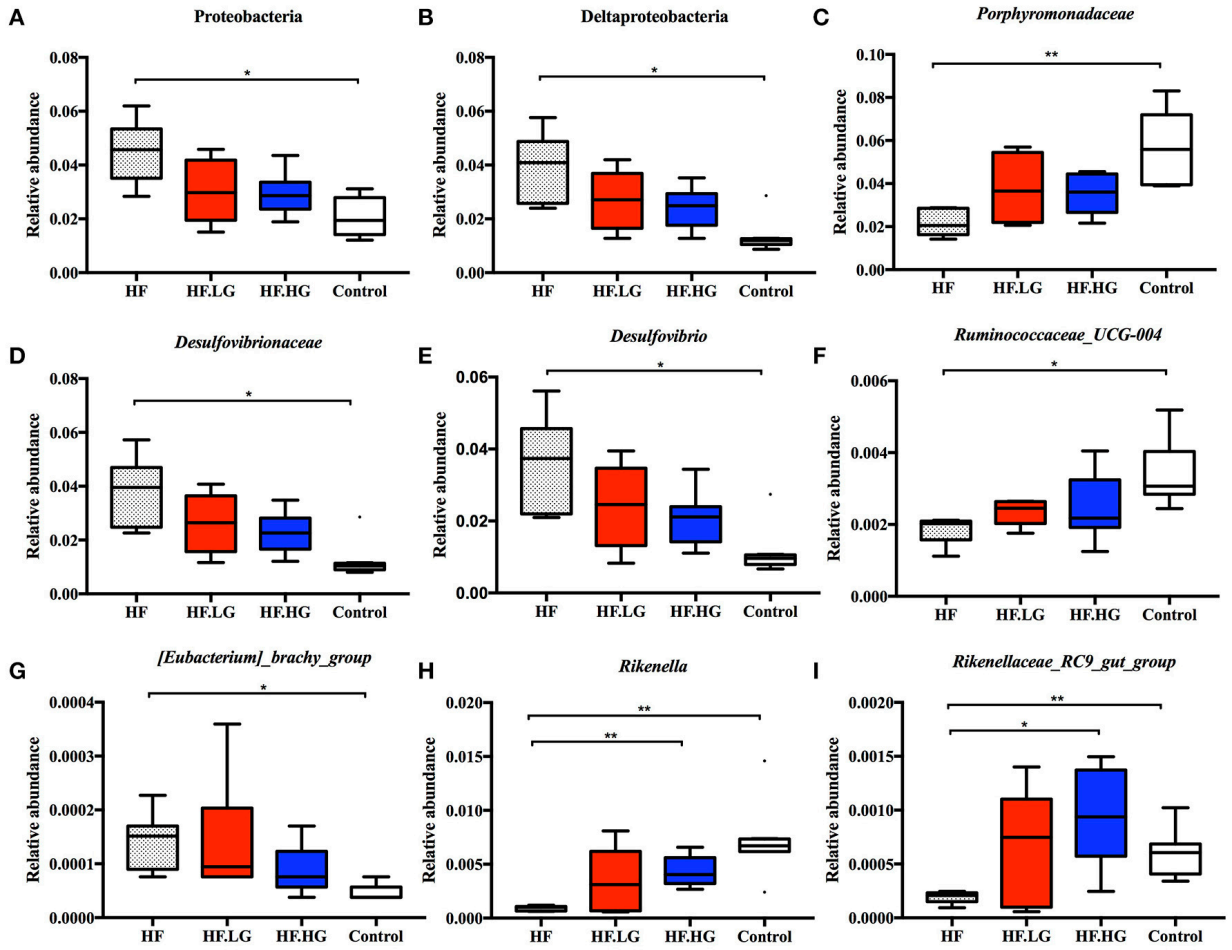
Relative abundance of bacterial taxa at different taxonomic levels in each group. (*n* = 6–8/group). **(A)** Proteobacteria; **(B)** Deltaproteobacteria; **(C)** *Porphyromonadaceae*; **(D)** *Desulfovibrionaceae*; **(E)** *Desulfovibrio*; **(F)** *Ruminococcaceae_UCG-004*; **(G)** *[Eubacterium]_brachy_group*; **(H)** *Rikenella*; and **(I)** *Rikenellaceae_RC9_gut_group*. HF, high-fat diet without genistein; HF. LG, high-fat diet with low-dose genistein; HF. HG, high-fat diet with high-dose genistein; Control, normal control diet. Data was analyzed by MetaStat. Mean values were significantly different between other group and the HF group: ^*^q < 0.05, ^**^q < 0.01.

**Figure 7:**
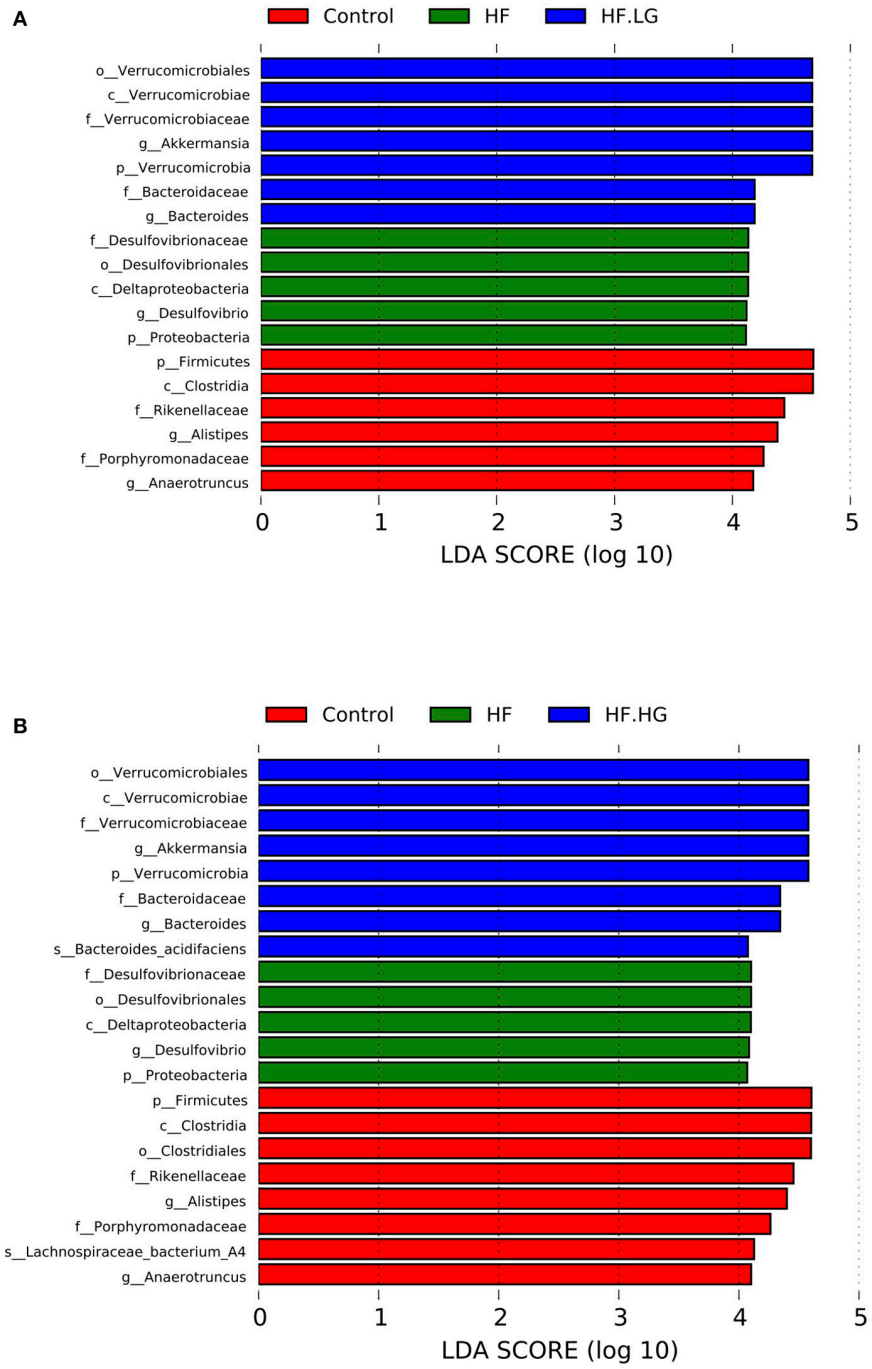
The LEfSe analysis of the different gut microbiota from the phylum level down to the species level (*n* = 6–8/group). **(A)** Differently enriched bacteria among the HF-HF.LG-Control group; and **(B)** Differently enriched bacteria among the HF-HF.HG-Control group. HF, high-fat diet without genistein; HF. LG, high-fat diet with low-dose genistein; HF. HG, high-fat diet with high-dose genistein; Control, normal control diet.

There were significant alterations in the composition of the intestinal microbial community among the four groups. MetaStat analysis showed that the phylum Proteobacteria, class Deltaproteobacteria, family *Desulfovibrionaceae* and genus *Desulfovibrio* were all significantly increased in the HF group compared with the Control group (*q* < 0.05) and were also relatively lower in the HF.LG group and HF.HG group compared to the HF group. However, there was a lower relative abundance of the family *Porphyromonadaceae*, genus *Ruminoccaceae_UCG-004*, genus *[Eubacterium]_brachy_group*, genus *Rikenella* and genus *Rikenellaceae_RC9_gut_group* in the HF group compared to the Control group (*q* < 0.05), all of which also tended to increase in female offspring at weaning after a maternal dietary genistein treatment in comparison with offspring from dams fed a high-fat diet without genistein. The genera *Rikenella* and *Rikenellaceae_RC9_gut_group*, both from the family *Rikenellaceae*, were significantly increased in the HF.HG group compared to the HF group and may play important roles in lipid metabolism (Figures 6A–I). Figure 7 lists significant microbiota changes from the phylum level to the species level, as performed by LEfSe. The phylum Firmucutes, class Clostridia, family *Rikenellaceae*, family *Porphyromonadaceae*, genus *Alistipes* and genus *Anaerotruncus* were significantly enriched in the control group. The phylum Proteobacteria, class Deltaproteobacteria, order Desulfovibrionales, family *Desulfovibrionaceae* and genus *Desulfovibrio* exhibited higher abundances in the HF group. Low-dose and high-dose genistein treatment both increased *Bacteroides* and the *Akkermansia* at the genus level (Figures 7A,B). By contrast, the species Bacteroides_acidifaciens was uniquely enriched in the HF.HG group (Figure 7B).

### Correlation analyses of the gut microbiota and glucose and lipid metabolic parameters

To assess associations between glucose and lipid metabolism and the gut microbiota in offspring, the AUC of the OGTT, insulin, HOMA-IR, TC, and TG levels were correlated with the intestinal bacterial relative abundance (Table 2). The AUC of the OGTT, fasting insulin levels, HOMA-IR and serum TC levels were positively correlated with the relative abundance of Proteobacteria at the phylum level and the class Deltaproteobacteria from the phylum Proteobacteria. However, the fasting serum insulin concentration and HOMA-IR were negatively correlated with the class Clostridia (phylum Firmicutes). At the family level, the AUC of the OGTT, insulin levels, HOMA-IR and serum TC levels were positively correlated with the abundance of *Desulfovibrionaceae* from the class Deltaproteobacteria. By contrast, the AUC of the OGTT, insulin levels, and HOMA-IR were all negatively correlated with the families *Porphyromonadaceae* and insulin levels, and HOMA-IR were both negatively correlated with *Rikenellaceae*, both of which belong to the phylum Bacteroidetes. At the genus level, the AUC of the OGTT, insulin and HOMA-IR were positively correlated with the genus *Desulfovibrio* from the phylum Firmicutes, but insulin and HOMA-IR were negatively correlated with the *Alistipes* and *Rikenella* from the phylum Bacteroidetes. In addition, HOMA-IR were also positively correlated with the species *Bacteroides_acidifaciens*. In regard to lipid metabolism, TC was positively correlated with the genus *Desulfovibrio*.

**Table 2:** Correlation analyses between relative abundance of bacterial taxa at different taxonomic levels and glucose and lipid metabolism parameters (*n* = 6–8/group).

**Metabolic index**	**Taxonomic level**	**Specific Taxon**	***r***	***p*-value**	**FDR**
AUC	phylum	Proteobacteria	0.59	0.0009	0.0033
	class	Deltaproteobacteria	0.59	0.0010	0.0028
	family	*Desulfovibrionaceae*	0.60	0.0008	0.0044
	family	*Porphyromonadaceae*	−0.52	0.0043	0.0095
	genus	*Desulfovibrio*	0.61	0.0005	0.0055
Insulin	phylum	Proteobacteria	0.50	0.0067	0.0082
	class	Clostridia	−0.52	0.0044	0.0081
	class	Deltaproteobacteria	0.51	0.0057	0.0090
	family	*Desulfovibrionaceae*	0.53	0.0041	0.0090
	family	*Porphyromonadaceae*	−0.58	0.0013	0.0143
	family	*Rikenellaceae*	−0.53	0.0036	0.0099
	genus	*Alistipes*	−0.53	0.0034	0.0125
	genus	*Rikenella*	−0.54	0.0029	0.0160
	genus	*Desulfovibrio*	0.51	0.0061	0.0084
	species	*Bacteroides_acidifaciens*	0.38	0.0460	0.0506
HOMA-IR	phylum	Proteobacteria	0.58	0.0013	0.0024
	class	Clostridia	−0.47	0.0108	0.0132
	class	Deltaproteobacteria	0.60	0.0007	0.0026
	family	*Desulfovibrionaceae*	0.62	0.0005	0.0055
	family	*Porphyromonadaceae*	−0.62	0.0005	0.0028
	family	*Rikenellaceae*	−0.50	0.0071	0.0111
	genus	*Alistipes*	−0.49	0.0083	0.0114
	genus	*Rikenella*	−0.59	0.0010	0.0022
	genus	*Desulfovibrio*	0.59	0.0009	0.0025
	species	*Bacteroides_acidifaciens*	0.46	0.0130	0.0143
TC	phylum	Proteobacteria	0.45	0.0161	0.0443
	class	Deltaproteobacteria	0.53	0.0037	0.0407
	family	*Desulfovibrionaceae*	0.51	0.0059	0.0325
	genus	*Rikenellaceae_RC9_gut_group*	−0.39	0.0382	0.0840
	genus	*Desulfovibrio*	0.46	0.0136	0.0499

*Spearman's correlations coefficients are listed for each taxonomy level. AUC, area under the curve; HOMA-IR, the homeostasis model assessment of insulin resistance; TC, total cholesterol; TG, triacylglycerol*.

## Discussion

It is well established that a poor maternal diet is an important factor in the development of metabolic disorders in offspring (29–32), which may have contributed to the current rapid increase in the prevalence of obesity and diabetes. Similarly, the present study also demonstrated that a maternal high-fat diet before pregnancy and during pregnancy and lactation could result in glucose intolerance, insulin resistance and higher serum levels of TC and TG in the early life of female offspring. It has been reported that genistein has anti-diabetic (13) and lipid metabolism improvement (33) functions, but the effects of genistein intake during pregnancy and lactation on glucose and lipid metabolism in offspring are poorly understood. In the present study, we explored the effects of maternal dietary genistein on glucose and lipid metabolism in female offspring at weaning. We found that maternal dietary low-dose genistein (0.25 g/kg diet) fully counteracted the detrimental effects of the maternal high-fat diet on glucose tolerance, circulating insulin, HOMA-IR and birth weight in female offspring. Moreover, disorders of the serum lipid profiles in offspring due to a maternal high-fat diet were prevented if dams were fed high-dose genistein (0.6 g/kg diet). The uterus and ovary index showed that the genistein intervention had no adverse effects on dams. These data indicate that maternal dietary genistein is pivotal for improving metabolic health in the early life of female offspring in a dose-dependent manner.

There might be changes in many organizations of the offspring that play important roles in the beneficial effects of maternal dietary genistein and the deleterious effects of a maternal high-fat diet on metabolic disorders in offspring. Given the vital role that the intestinal microbial community play in metabolic health, we hypothesized that the intestinal microbiota of the offspring changed. Indeed, our results showed that maternal high-fat feeding leaded to significant alterations in the overall structure and composition of the intestinal microbial community and that maternal genistein intake reversed these detrimental effects. The present study showed that Proteobacteria, Deltaproteobacteria, Desulfovibrionales, *Desulfovibrionaceae* and *Desulfovibrio* significantly increased and were positively correlated with glucose and lipid metabolic parameters, whereas *Porphyromonadaceae, Ruminoccaceae_UCG-004, [Eubacterium]_brachy_group, Rikenella* and *Rikenellaceae_RC9_gut_group* significantly decreased and were negatively correlated with glucose and lipid metabolic parameters in female offspring from high-fat fed dams compared to those in female offspring of Control group dams. As previously shown, high-fat feeding resulted in significantly increased abundance of Proteobacteria and *Desulfovibrionaceae*. Tomas et al. (34) found that a high-fat diet altered the composition of the fecal and cecal microbial community even after 30 d of consumption and that the relative abundance of Proteobacteria significantly increased. In Proteobacteria, the main increase was in class Deltaproteobacteria (Desulfovibrionales order, *Desulfovibrionaceae* family). Meanwhile, the physical integrity of the epithelial barrier was disrupted and intestinal permeability was increased. Similarly, another previous study showed that the intestinal microbiota of wild-type mice switched to high-fat diets has changed a lot, including an increase in Proteobacteria. The main group of Proteobacteria increased in relative abundance, as did the class DeltaProteobacteria and genus *Desulfovibrio* (35). Several genera belonging to the *Desulfovibrionaceae* family are considered to be opportunistic pathogens and have been linked to some inflammatory diseases (36, 37). These genera produce endotoxins and have the capacity to reduce sulfate to H_2_S (38), thereby damaging the intestinal barrier (39). Maternal dietary genistein decreases the abundance of these bacteria and reverses their detrimental effects on metabolism. In addition, Li et al. (40) analyzed the effect of the antibiotic azithromycin on the gut microbiota and adipogenesis in mice and found that the abundance of *Rikenella* was significantly lower in the azithromycin group and was associated with a higher body weight and larger percentage of body fat. Another human study also indicated that the abundance of *Rikenellaceae*, along with other bacterial components, contributed to a lean body type (41). In the present study, maternal dietary high-dose genistein significantly increased the abundance of *Rikenella* and *Rikenellaceae_ RC9_ gut_group* and improved the levels of TG and TC in female offspring at weaning. Thus, the genus of *Rikenella* and *Rikenellaceae_ RC9_ gut_group* might play crucial roles in the improved lipid metabolism by high-dose genistien.

In the current study, at the genus level, maternal dietary genistein (including HF.LG and HF.HG) significantly enriched *Bacteroides* and *Akkermansia*. Several human studies have shown that the relative abundance of *Bacteroides* was decreased in type 2 diabetes patients in comparison to normal control subjects (42, 43). A fiber-rich macrobiotic Ma-Pi 2 diet increased the abundance of propionate producers (*Bacteroides*) (42). In addition, another obese mice study showed that resveratrol improved glucose tolerance while increasing the relative abundance of *Bacteroides* (44). Our study found that *Bacteroides* was enriched in female offspring from genistein fed dams and might play crucial roles in negating the deleterious effects of a poor maternal diet on the metabolism of offspring. The beneficial effects of maternal genistein intake on the metabolism of offspring were also associated with a significant enrichment in the relative abundance of *Akkermansia*, which could maintain the mucus layer thickness and reduce leakage of LPS and intestinal permeability (45).

To our knowledge, this is the first study that showed that the phytoestrogen genistein exerts a significant effect on the abundance of *Akkermansia* in the gut microbial commuity of an animal model of maternal high-fat diet-induced offspring metabolic disorders. Recently, it has been reported that administration of polyphenols was also associated with an increased abundance of *Akkermansia* in both human and animal studies (46, 47). Moreover, an increase in the gut proportion of this bacterium has also been associated with the beneficial effects of the anti-diabetic drug metformin and gastric bypass surgery on metabolism (48, 49). Although we have not directly determined the causality between the increased proportion of *Akkermansia* and the improvement of glucose and lipid metabolism in offspring from genistein intake high-fat fed dams, it has been reported that oral administration of *Akkermansia* reverses the metabolic abnormalities induced by a high-fat diet (45) and also mimics the antidiabetic effects of metformin in diabetic mice (48). More importantly, our results indicated that the increase in *Akkermansia* associated with genistein might be sufficient to improve metabolic disorders in offspring induced by a maternal high-fat diet without significant alterations in the proportions of Firmicutes and Bacteroidetes.

In addition, LEfSe analysis showed that the species *Bacteroides_acidifaciens* was uniquely enriched in the HF.HG group. Renouf et al. (50) first identified fecal microbes that were responsible for the degradation of isoflavone using molecular genetic techniques and demonstrated that *Bacteroides_acidifaciens* increased the disappearance of isoflavone genistein in human fecal incubating under anaerobic and nutrient-rich conditions, which indicated that *Bacteroides_acidifaciens* played a role in the metabolism of genistein in the intestine. Thus, the enrichment of *Bacteroides_acidifaciens* in the HF.HG group increased the degradation of isoflavones genistein. Correlation analysis showed that the relative abundance of *Bacteroides_acidifaciens* was positively related with HOMA-IR, which clarified the differences of the effects of maternal low-dose genistein and high-dose genistein on the glucose metabolism and insulin sensitivity in the offspring.

In summary, maternal dietary genistein provided before and during pregnancy and lactation significantly improved the metabolism of female offspring in early life and compensated for the detrimental effects of a maternal high-fat diet. The improvement in glucose and lipid metabolism is associated with the alterations in the gut microbiota of offspring. This is the first study to report the role that the gut microbiota plays in the effects of maternal dietary genistein on glucose and lipid metabolism in female offspring. However, our study only analyzed the relationship between gut microbiota and metabolism in offspring and the causality is still needed to be further explored. Furthermore, only female offspring was studied in this study. The effects of maternal dietary genistein on metabolic health of male offspring is worth to be studied in the future. Our results suggest that the provision of maternal dietary genistein before pregnancy and during pregnancy and lactation may be an important tool for combating obesity and diabetes in offspring.

## Data availability

The datasets supporting the conclusions of this manuscript are available from the corresponding author on reasonable request.

## Author contributions

XX and JZ designed the experiments. LZ, XW, MD, RL, and XZ performed the experiments. LZ analyzed the data and wrote the original draft. XX, JZ, QZ, ML, and MY reviewed the manuscript. All of the authors had final approval of the submitted version.

### Conflict of interest statement

The authors declare that the research was conducted in the absence of any commercial or financial relationships that could be construed as a potential conflict of interest.
