# Supplementary material for: Improved Glucose and Lipid Metabolism in the Early Life of Female Offspring by Maternal Dietary Genistein Is Associated With Alterations in the Gut Microbiota
Source: Front Endocrinol (Lausanne). 2018 Sep 4;9:516. doi: 10.3389/fendo.2018.00516 (PMC6131301; doi:10.3389/fendo.2018.00516)
Supplement: Supplementary file 1 [file Table_1.docx]

Supplementary Material

Improved Glucose and Lipid Metabolism in Early Life of Female Offspring by Maternal Dietary Genistein Is Associated With Alterations in the Gut Microbiota

**Liyuan Zhou^1^, Xinhua Xiao^1^*, Qian Zhang^1^, Jia Zheng^1^, Ming Li^1^, Miao Yu^1^, Xiaojing Wang^1^, Mingqun Deng^1^, Xiao Zhai^1^, Rongrong Li^1^**

^1^Key Laboratory of Endocrinology, Translational Medicine Center, Ministry of Health, Department of Endocrinology, Peking Union Medical College Hospital, Peking Union Medical College, Chinese Academy of Medical Sciences, Beijing, China

# * Correspondence: Xinhua Xiao, [xiaoxh2014@vip.163.com](mailto:xiaoxh2014@vip.163.com)

**Table S1.** The nutritional compositions of four types of diet. HF, high-fat diet without genistein; HF. LG, high-fat diet with low-dose genistein; HF. HG, high-fat diet with high-dose genistein; Control, normal control diet.

| **Ingredients** | **HF.LG(g)** | **HF.HG(g)** | **HF(g)** | **Control(g)** |
| --- | --- | --- | --- | --- |
| Casein | 258 | 258 | 258 | 200 |
| L-Cystine | 4 | 4 | 4 | 3 |
| Corn Starch | 0 | 0 | 0 | 397 |
| Maltodextrin | 162 | 162 | 162 | 132 |
| Sucrose | 89 | 89 | 89 | 100 |
| Cellulose | 65 | 65 | 65 | 50 |
| Soybean Oil | 0 | 0 | 0 | 0 |
| Corn Oil | 32 | 32 | 32 | 70 |
| t-Butylhydroquinone | 0 | 0 | 0 | 0.014 |
| Mineral Mix S10026 | 13 | 13 | 13 | 0 |
| MinarelMix S10022G | 0 | 0 | 0 | 35 |
| Vitamin Mix V10001 | 13 | 13 | 13 | 0 |
| Vitamin Mix V10037 | 0 | 0 | 0 | 10 |
| Choline Bitartrate | 2.6 | 2.6 | 2.6 | 2.5 |
| Lard | 316.6 | 316.6 | 316.6 | 0 |
| DiCalcium Phosphate | 16.8 | 16.8 | 16.8 | 0 |
| Calcium Carbonate | 7 | 7 | 7 | 0 |
| Potassium Citrate, 1 H2O | 21 | 21 | 21 | 0 |
| FD&C Blue Dye #1 | 0.06 | 0.06 | 0.06 | 0 |
| Genistein | 0.25 | 0.6 | 0 | 0 |
| Total | 1000 | 1000 | 1000 | 1000 |

**Table S2.** Comparison of estimator indices of alpha diversity. Data are expressed as means ± S.E.M (n=6-8/group). HF, high-fat diet without genistein; HF. LG, high-fat diet with low-dose genistein; HF. HG, high-fat diet with high-dose genistein; Control, normal control diet.

| **Estimators** | **HF.LG** | **HF.HG** | **HF** | **Control** |
| --- | --- | --- | --- | --- |
| Simpson | 0.960±0.009 | 0.965±0.003 | 0.972±0.003 | 0.974±0.001 |
| Chao1 | 376.5±12.5 | 383.8±5.4 | 395.5±11.6 | 392.2±9.9 |
| Shannon | 5.94±0.14 | 5.98±0.07 | 6.18±0.08 | 6.25±0.05 |

**Figure S1.** Rarefaction Plot of Simpson, Chao1 and Shannon index.
